# Suppressing interferences of EIT on synchronous recording EEG based on comb filter for seizure detection

**DOI:** 10.3389/fneur.2022.1070124

**Published:** 2022-12-02

**Authors:** Lei Wang, Wenjing Zhu, Rong Wang, Weichen Li, Guohua Liang, Zhenyu Ji, Xiuzhen Dong, Xuetao Shi

**Affiliations:** ^1^Institute of Medical Research, Northwestern Polytechnical University, Xi'an, China; ^2^School of Life Sciences, Northwest University, Xi'an, China; ^3^Department of Medical Electronic Engineering, School of Biomedical Engineering, The Fourth Military Medical University, Xi'an, China

**Keywords:** epilepsy, electrical impedance tomography, EEG signal, low-frequency interference, comb filter

## Abstract

**Background and objective:**

The purpose of this study was to eliminate the interferences of electrical impedance tomography (EIT) on synchronous recording electroencephalography (EEG) for seizure detection.

**Methods:**

The simulated EIT signal generated by COMSOL Multiphysics was superimposed on the clinical EEG signal obtained from the CHB-MIT Scalp EEG Database, and then the spectrum features of superimposed mixed signals were analyzed. According to the spectrum analysis, in addition to high-frequency interference at 51.2 kHz related to the drive current, there was also low-frequency interference caused by switching of electrode pairs, which were used to inject drive current. A low pass filter and a comb filter were used to suppress the high-frequency interference and low-frequency interference, respectively. Simulation results suggested the low-pass filter and comb filter working together effectively filtered out the interference of EIT on EEG in the process of synchronous monitoring.

**Results:**

As a result, the normal EEG and epileptic EEG could be recognized effectively. Pearson correlation analysis further confirmed the interference of EIT on EEG was effectively suppressed.

**Conclusions:**

This study provides a simple and effective interference suppression method for the synchronous monitoring of EIT and EEG, which could be served as a reference for the synchronous monitoring of EEG and other medical electromagnetic devices.

## Introduction

Epilepsy is a nervous system disease closely related to changes in cerebral blood flow. Regional cerebral blood flow (rCBF) that occurs during seizure evolution has indicated a significant clinical value (1, 2). About one-third of epileptic patients suffering from drug-resistant (or medically intractable) epilepsy (3) have their daily lives disrupted by the occurrence of sudden seizures (4); thus, information about rCBF is extremely important. This is because surgery is an optimal treatment for patients with drug-resistant epilepsy (5, 6), and the effectiveness of surgery depends heavily on the accuracy of localization (7). However, the epileptogenic zone (EZ) is a theoretical construct, and to date, there is no established marker that definitively determines its location and extent (8, 9). Many surgical candidates undergo invasive intracranial electroencephalography (EEG) recording, but this technique has several limitations, in that it is invasive, high-cost, and offers limited spatial sampling that may lead to misleading conclusions (10, 11). Given the lack of an unambiguous marker for the EZ and the limitations of invasive intracranial EEG, the presurgical delineation of the EZ is complicated and often unsuccessful. As a result, a large proportion of patients who undergo epilepsy surgical resection for epilepsy treatment continue to have seizures after the surgery (12–14).

Techniques based on functional imaging, such as functional magnetic resonance imaging (fMRI) (15, 16), single-photon-emission CT (SPECT) (17) and positron emission tomography (PET) (18), have been developed to evaluate the location of the EZ as part of the pre-surgical work-up of epilepsy patients by assessesing hemodynamics of the brain such as cerebral blood volume and cerebral blood flow. However, SPECT and PET are nuclear imaging procedures that acquire images based on radiopharmaceuticals. Although MRI does not emit ionizing radiation, it does employ a strong magnetic field that extends beyond the machine and exerts very powerful forces on objects made of iron, some types of steel, and other magnetizable objects. All of these techniques are impractical for continuous monitoring. Moreover, these techniques have a long detection time, high cost (19) and locating area beyond the EZ (20), all of which are limitations to locating the EZ. Therefore, 25–33% of patients still “fail” surgical resection (21), and ~40% of patients continue their anti-epileptic medications even after surgery (22). To improve the safety and efficacy of surgical treatment for epilepsy, it is necessary to identify and validate reliable biomarkers that can determine the extent and location of the EZ with high precision and accuracy (23).

Electrical impedance tomography (EIT) is a functional imaging technique that can reconstruct images of electrical impedance within the body associated with functional changes in tissues or organs (24). Compared with CT, MRI, and other traditional medical imaging technologies, the advantages of EIT are that it is safer, non-invasive, and does not produce radiation. Plus, it is portable, which enables continuous bedside monitoring of various physiological and pathological process. EIT has already been used successfully in breast cancer detection (25, 26) and lung (27, 28), gastric (29), brain function imaging (30, 31), and in many other medical areas. It could also be used to image impedance changes related to blood flow (32). Impedance changes due to blood flow during rest and hyperemia could be measured by EIT in venous occlusion experiments. It was worthwhile to investigate EIT for the study of disease detection or pathological monitoring related to blood flow.

In light of the above, the following question arises: Is it possible to locate the EZ based on the impedance changes caused by epileptic seizures associated with rCBF by EIT? A group of researchers has been working on this problem since 1994. EIT has previously been proposed as a complementary tool to the existing EEG recording equipment for improving the preoperative localization foci, with no additional risks for patients (33–37). Three-dimensional (3D) EIT developed in recent years (38–40) has the potential to improve localized distinguishability of EZ by adding volumetric resolution. EIT can be used in presurgical assessment simultaneously with EEG to provide more changes related to epileptic seizures and facilitate localization of the EZ. However, each impedance measurement in EIT is obtained by injecting current at about a few milliamps at tens of kilohertz, which is well above the EEG band and generate artifacts in the EEG. Therefore, the EEG signal is completely obscured by simultaneous EIT recording and the epileptic EEG waves cannot be recognized. Some efforts have been made to remove the artifact induced in the EEG by simultaneous EIT recording by using dedicated hardware filter and software filter and separate EIT and EEG electrodes (24, 36, 41). This method requires advanced EEG recording to establish the noise template. However, due to differences between the template and actual artifact waveforms, the robustness of this method is not satisfactory. Furthermore, for better location of the EZ, EIT and EEG should use the same electrodes rather than separate electrodes. Multi-frequency electrical impedance tomography (MFEIT) has been used to avoid interference of EIT to EEG without switching injection electrode pairs (42–44), but the greater complexity of the current source hardware, signal processing, stray capacitance and reduced EIT protocol could reduce the measurement accuracy and speed (43).

This study investigated the spectral feature of the interference of EIT in EEG to remove this interference when using the same electrodes for EIT and EEG. The simulated EIT signal generated by COMSOL Multiphysics was superimposed on the clinical EEG signal obtained from the CHB-MIT Scalp EEG Database, and then the spectral features of superimposed mixed signals were analyzed. According to the spectral analysis, in addition to a high-frequency interference at 51.2 kHz related to the drive current, there was also a low-frequency interference caused by switching electrode pairs, which was used to inject drive current. A low pass filter and a comb filter were used to suppress high-frequency interference and low-frequency interference, respectively. Finally, the filtering effect was verified by Pearson correlation between the filtered EEG and the raw EEG.

## Method

### Simulated EIT recording

**Figure 1:**
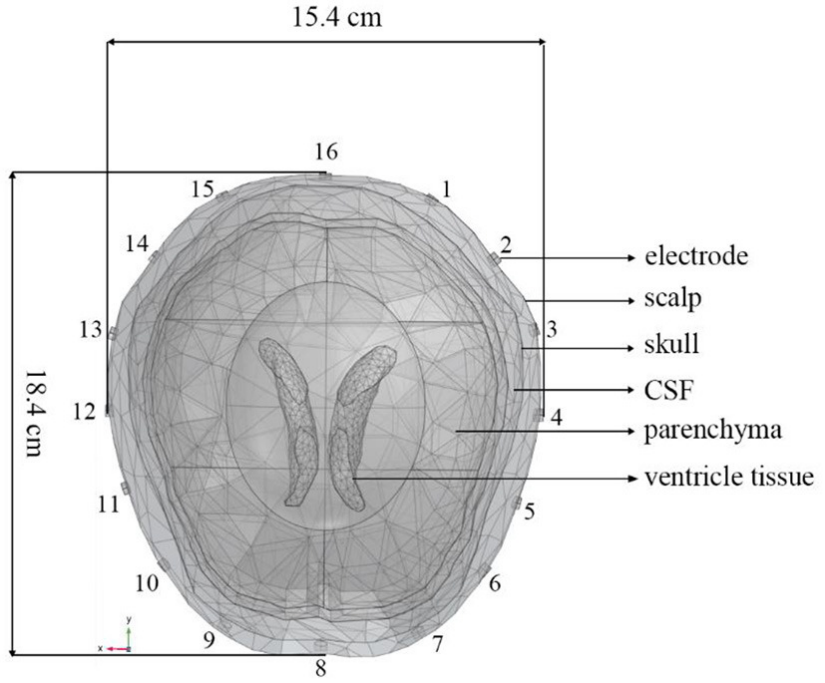
A 3D finite element head model including scalp, skull, CSF, brain parenchyma, and ventricle tissues generated by COMSOL Multiphysics. The circumference, length, and width of the head model are, respectively 56.2, 18.4, and 15.4 cm. The 16 electrodes were numbered counterclockwise.

A 3D finite element (FE) head model (45) established by COMSOL Multiphysics 4.4 (Comsol Group, Sweden) was used in our simulation experiments. It consisted of brain tissues including scalp, skull, cerebrospinal fluid (CSF), brain parenchyma, and ventricle tissues (Figure 1). The circumference, length, and width of the head model were, respectively 56.2, 18.4, and 15.4 cm. A total of 16 electrodes were evenly placed on the model and numbered in counterclockwise order. A sinusoidal current of 1 mA, 51.2 kHz was injected into the model through a pair of excitation electrodes. The conductivity parameters of the scalp, skull, CSF, brain parenchyma, and ventricle tissues are set. According to the electromagnetic field boundary conditions in the positive problem of electrical impedance imaging, the potential distribution of the entire head region is solved, and the potential values of all the remaining measured electrodes on the head are obtained.

Under certain measurement accuracy of the EIT system, the impedance information, signal-to-noise ratio, and imaging quality measured under different drive patterns will be different (46, 47), and the interference to EEG signals will change accordingly. Thus, it is necessary to develop a method suitable for removing the interference from EIT systems with different drive patterns. Drive pattern refers to the way in which EIT excitation current is injected. Polar drive pattern, adjacent drive pattern, and Avis-Barber cross drive pattern are the three commonly used patterns to inject excitation current in EIT. In the polar drive pattern, a pair of electrodes with an angle of 180° are used for injecting excitation current. In the adjacent drive pattern, a pair of adjacent electrodes are used for injecting excitation current. In the Avis-Barber cross drive pattern, a pair of electrodes with an angle of 90 degrees are used for injecting excitation current. The boundary voltages between all the remaining adjacent electrode pairs are measured for the three drive patterns. In light of this, the spectral features of EIT under these three common drive patterns were analyzed.

### Clinical EEG data

**Figure 2:**
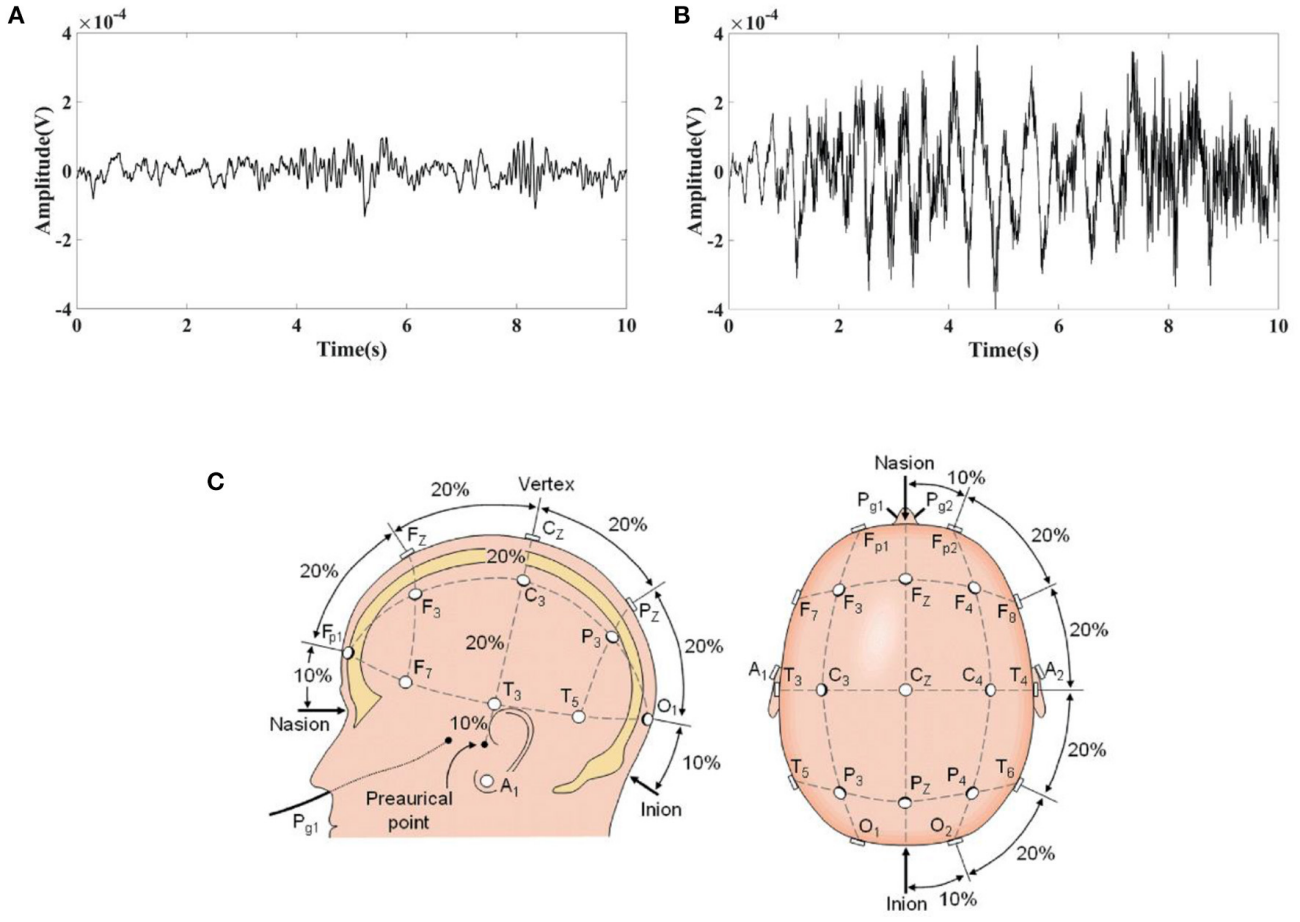
Typical EEG fragments obtained from the CHB-MIT Scalp EEG Database and the electrode positions used for these recordings. **(A)** 10 s Normal EEG; **(B)** 10 s Epileptic EEG; **(C)** The International 10–20 system used for recording EEGs in the CHB-MIT Scalp EEG Database seen from left and above the head.

Clinical EEG data were obtained from the CHB-MIT Scalp EEG Database (48). This database consists of EEG recordings taken at Children's Hospital Boston from pediatric subjects with intractable seizures. In the development of this database, subjects were monitored for up to several days following withdrawal of anti-seizure medication to characterize their seizures and assess their candidacy for surgical intervention. All of the signals were sampled at 256 samples per second with 16-bit resolution. Randomly selected 10 s normal EEG and epileptic EEG were shown in Figures 2A,B, from which obvious difference can be seen in the EEG waves between normal and epileptic state. The International 10–20 system of EEG electrode positions and nomenclature was used for these recordings (Figure 2). Bipolar arrangements were used in EEG recording. The numbers “10” and “20” refer to the fact that the distances between adjacent electrodes are either 10% or 20% of the total front- back or right-left distance of the skull. Each site has a letter to identify the lobe and a number to identify the hemisphere location. (https://www.diytdcs.com/2012/07/1020-system-electrode-distances/).

### Spectral feature analysis of mixed signal

**Figure 3:**
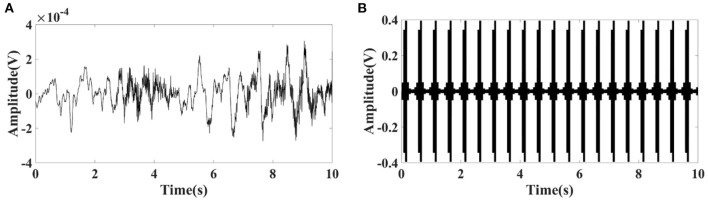
**(A)** Randomly selected 10 s raw epileptic EEG from the CHB-MIT Scalp EEG Database, and **(B)** its waveform interfered by simultaneously recorded EIT under polar drive pattern with one frame per second.

The EIT signal simulated by COMSOL Multiphysics was superimposed on the EEG signal obtained from the CHB-MIT Scalp EEG Database. The superimposed signal was used to simulate the EEG signals interfered by EIT when EIT and EEG were recorded simultaneously. It can be seen from Figures 1, 2 that the number and locations of electrodes in EIT are inconsistent with those in the EEG system. Fp2 and F8 in EEG are close to the number 2 and 3 electrodes in EIT. Data collected by these electrodes are superposed in this simulation study. Electrode location differences between the two systems might not change the results in the simulation study. Figure 3 showed a randomly selected 10 s raw epileptic EEG and its waveform interfered by EIT. The EEG signal was completely lost within the high-frequency interference from simultaneously recorded EIT.

**Figure 4:**
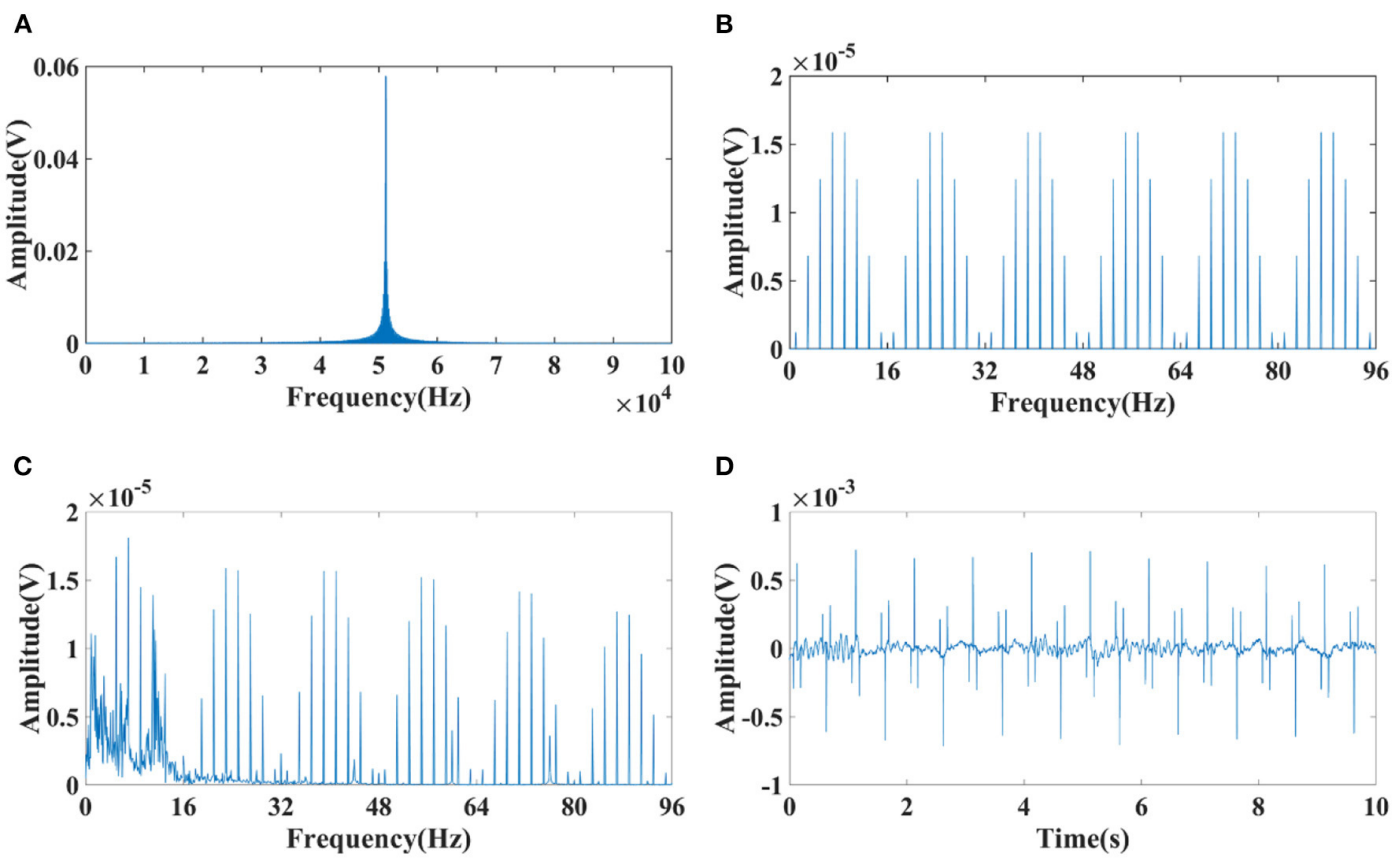
Spectrum diagram of EIT and interfered EEG signals. **(A)** High–frequency component at 51.2 kHz of EIT; **(B)** Low–frequency component with a period of about the duration of electrode switching (62.5 ms, 16 Hz, under a polar drive pattern with one frame per second (1 fps)); **(C)** Frequency spectrum of the interfered EEG signals suppressed after low–pass filtering; **(D)** Time domain of the interfered EEG signals suppressed after low–pass filtering.

Spectrums of EIT was analyzed to suppress the interference of EIT on EEG. There was a high frequency component at 51.2 kHz, which consistent with the carrier frequency of 51.2 kHz applied in EIT (Figure 4A). A periodic low–frequency component with a period of about the duration of electrode switching [62.5 ms, 16 Hz, under a polar drive pattern with one frame per second (1 fps)] was also found in the EIT signal (Figure 4B). A low–pass filter with a cutoff frequency of 100Hz was selected to filter out high–frequency interference at 51.2 kHz (Fs = 800,000, Fc = 100 Hz, Order *N* = 2, Butterworth filter). The reason why 100 Hz was chosen as the cut-off frequency was that EEG signals of clinical significance are mostly in the frequency range of 0.5–100 Hz (49), including typical epileptic EEG such as spike waves, sharp waves, spike (or sharp) and slow waves. However, the low–frequency interference (LFI) of EIT on EEG could not be suppressed after low–pass filtering. The interference of low-frequency components still can be seen in the frequency domain (Figure 4C) and time domain (Figure 4D) of the interfered EEG signals.

**Figure 5:**
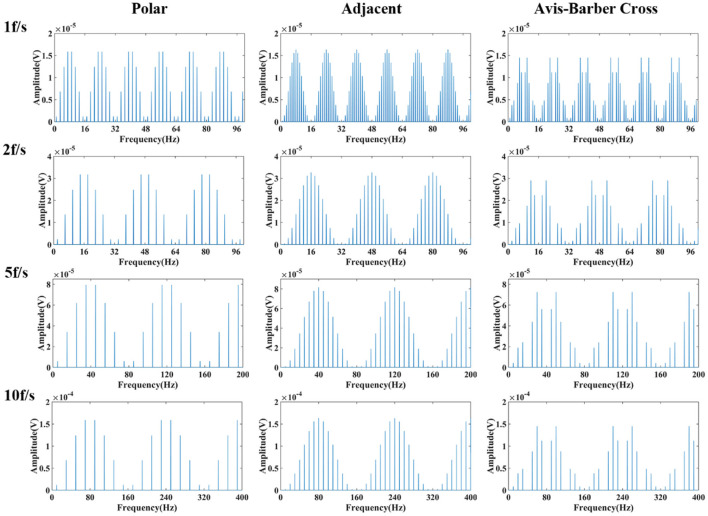
Frequency spectrum of low–frequency component of EIT under different drive patterns and acquisition frame rates. From left to right are the low–frequency spectra under the polar, adjacent, and Avis–Barber cross drive patterns. From top to bottom are the low–frequency spectra with acquisition frame rate of 1, 2, 5 and 10 frame.

Since the low–frequency component is related to the duration of electrode switching, which might be correlated with the drive pattern and acquisition frame rate of the EIT system, the spectral characteristics of the low–frequency component generated at different acquisition frame rates (1, 2, 5, 10 fps) in three commonly used drive patterns, namely the adjacent, polar, and Avis-barber cross drive patterns, were analyzed. As shown in Figure 5, the acquisition frame rate affects the periodic frequency of the low-frequency component, and the drive pattern affects the pulse waveform and spectrum distribution in each frequency period. When the acquisition frame rate was 1 fps, 2 fps, 5 fps and 10 fps, the frequency period was 16 Hz, 32 Hz (2^*^16 Hz), 80 Hz (5^*^16 Hz) and 160 Hz (10^*^16 Hz), respectively. In each period of the low–frequency component, the pulse number was largest with adjacent drive pattern, and least with polar drive pattern.

### Comb filter design

The low–pass filter with a cutoff frequency of 100 Hz, which can suppress the high–frequency interference at 51.2 kHz of EIT on EEG, but not suppress the low–frequency interference lower than 100 Hz. Upon close inspection of Figure 5, it can be seen that the low–frequency component resembles teeth in a comb, so it might be attenuated with the narrow and deep notches of comb filtering (50). A comb filter can be used to filter a periodic noise buried in signal, provided the teeth of the comb coincide with the periodic harmonics (51). As a physical tool, it has been used to improve the signal-to-noise ratio in biological signal detection (51, 52). To study the effectiveness of the comb filter, parameters of the comb filter were designed for four acquisition frame rates (1, 2, 5, 10 fps) under three drive patterns (Table 1). Frequency of sample, order, bandwidth and attenuation pass (Apass) are the key parameters that affect the filtering effect of comb filter. Theoretically, the larger these parameters are, the better the filtering effect will be. However, in practice, the filter design should take the filtering effect into account, and also avoid the signal distortion and signal delay. The sampling frequency was set at 300 Hz, similar to the EEG sampling rate, to balance the noise and signal delay. The order N of the comb filter defines the number of notches. In this study, N depends on the acquisition frame rate of EIT so that the notches of the comb filter coincide with the harmonics of the low–frequency interference. Bandwidth and Apass determine the ability of the comb filter to separate and attenuate the specified frequencies. Too large bandwidth and Apass will not only filter the interference but also cause the loss of EEG signal. On the contrary, too small bandwidth and Apass will not completely filter the interference. So the optimal bandwidth and Apass were determined by the actual filtering effect in this study.

**Table 1:** Design of comb filter under different drive patterns and acquisition frame rates.

**Drive Pattern**	**Acquisition frame rate (fps)**	**Frequency of sample (Fs/Hz)**	**Order (N)**	**Bandwidth (Bw/dB)**	**Attenuation pass (Apass/dB)**
Polar	1	300	300	0.1	0.1
	2	300	150	0.1	0.1
	5	300	60	0.15	0.1
	10	300	30	0.3	0.05
Adjacent	1	300	300	0.1	0.1
	2	300	150	0.1	0.1
	5	300	60	0.15	0.1
	10	300	30	0.3	0.05
Avis-Barber Cross	1	300	300	0.1	0.1
	2	300	150	0.1	0.1
	5	300	60	0.15	0.1
	10	300	30	0.3	0.05

## Results

### Low–pass filtering effect

**Figure 6:**
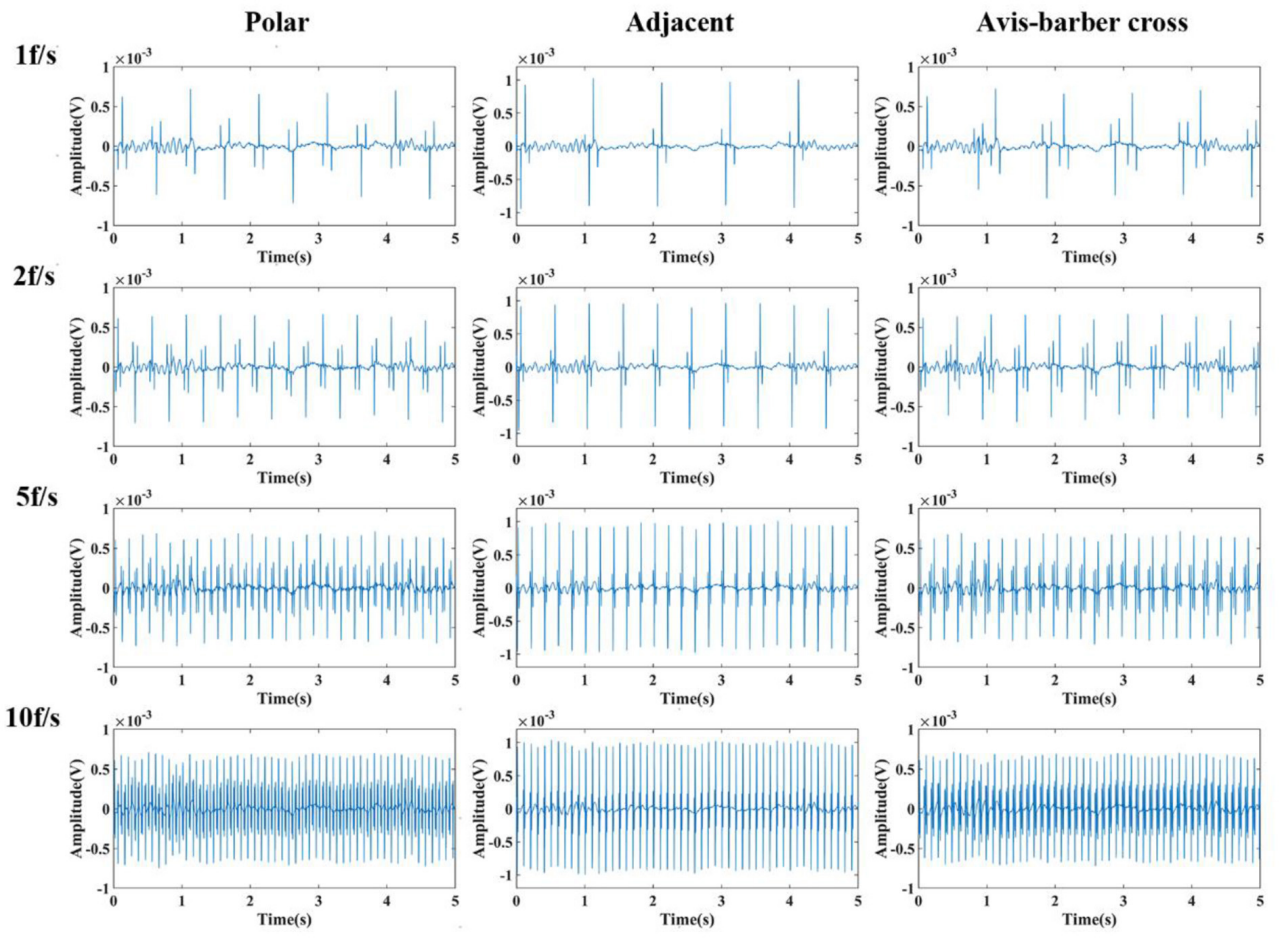
Low-pass filtered EEG after interfered by EIT with different drive patterns and acquisition frame rates. From left to right are the low-pass filtered EEG after interfered by EIT with the polar, adjacent, and Avis–Barber cross drive patterns. From top to bottom are the low-pass filtered EEG after interfered by EIT with acquisition frame rate of 1, 2, 5 and 10 frame.

**Figure 7:**
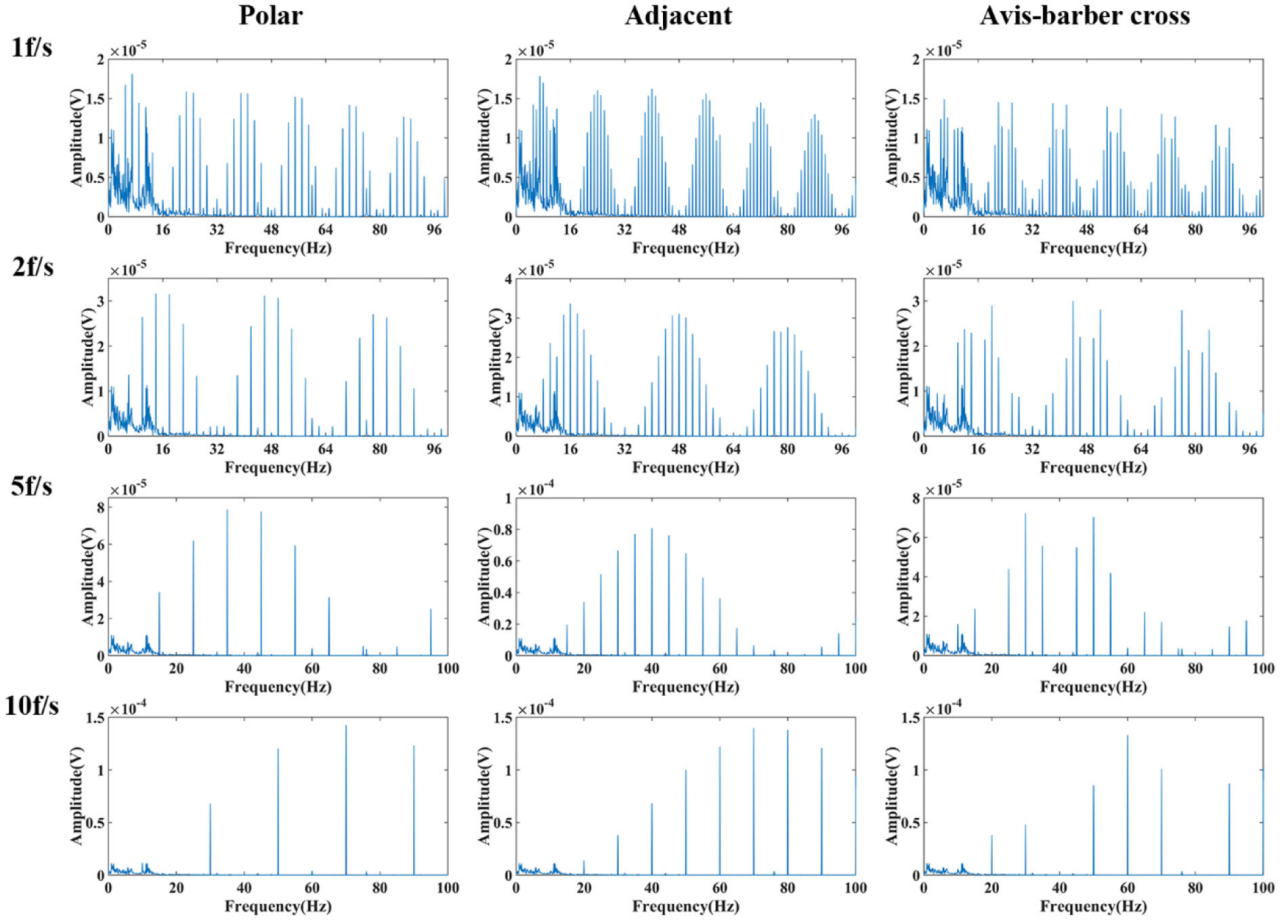
Frequency spectrum of low-pass filtered EEG after interfered by EIT with different drive patterns and acquisition frame rates. From left to right are the low-pass filtered EEG after interfered by EIT with the polar, adjacent, and Avis–Barber cross drive patterns. From top to bottom are the low-pass filtered EEG after interfered by EIT with acquisition frame rate of 1, 2, 5 and 10 frame.

After low–pass filtering, the high–frequency interference from EIT was completely suppressed, but periodic low–frequency interference still exists and the EEG waves cannot be identified (Figures 6, 7). The faster the acquisition frame rate, the shorter the low–frequency interference period, and the more difficult to distinguish EEG signal. Therefore, an additional filtering method was necessary.

### Comb filtering effect

**Figure 8:**
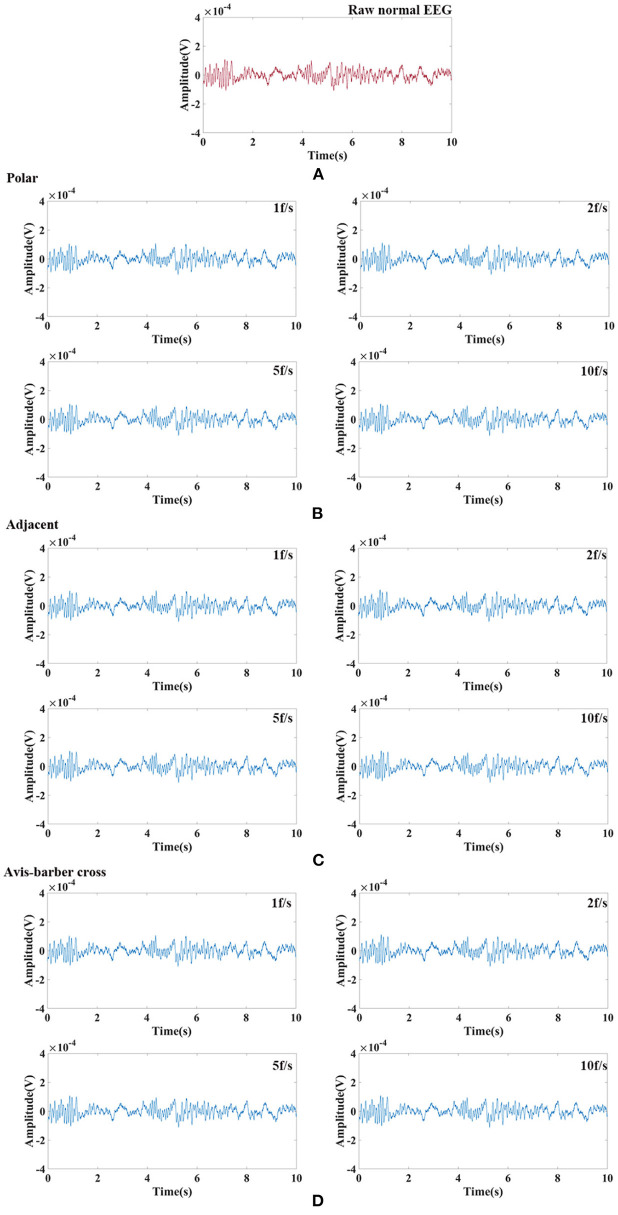
Raw normal EEG signals and its waveform filtered by low–pass filtering and comb filtering after interfered by EIT. **(A)** Raw normal EEG segment. **(B)** Low–pass and comb filtered EEG after interfered by EIT with the polar drive pattern and different acquisition frame rates; **(C)** Low–pass and comb filtered EEG after interfered by EIT with the adjacent drive pattern and different acquisition frame rates; **(D)** Low–pass and comb filtered EEG after interfered by EIT with the Avis–barber cross drive pattern and different acquisition frame rates.

**Figure 9:**
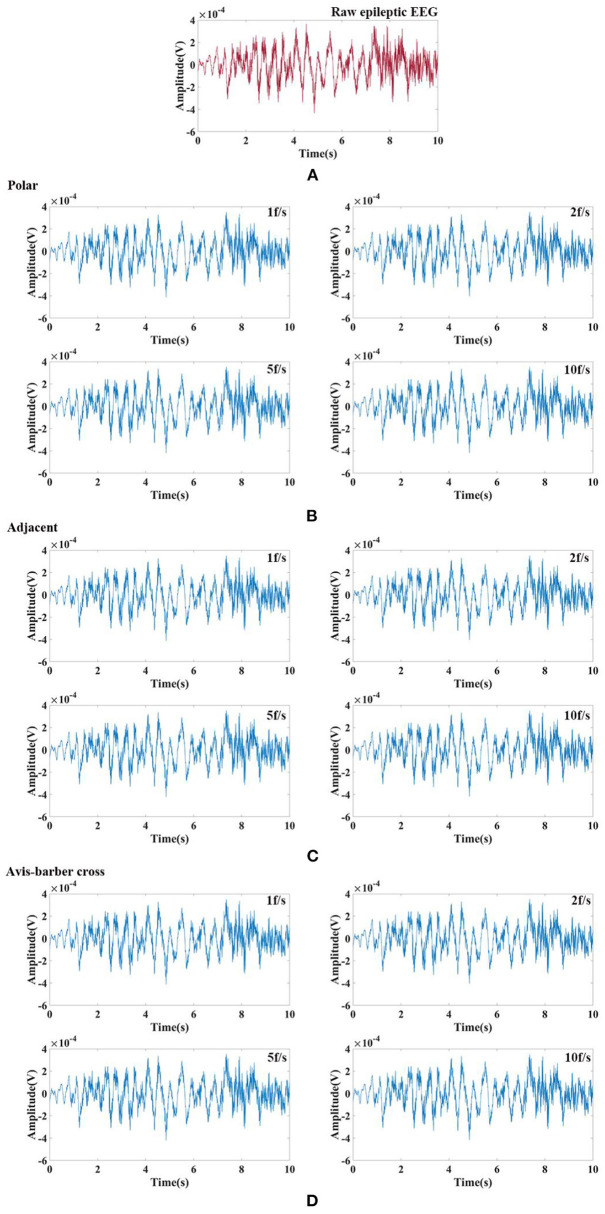
Raw epileptic EEG signals and its waveform filtered by low–pass filtering and comb filtering after interfered by EIT. **(A)** Raw epileptic EEG segment; **(B)** Low–pass and comb filtered epileptic EEG after interfered by EIT with the polar drive pattern and different acquisition frame rates; **(C)** Low–pass and comb filtered epileptic EEG after interfered by EIT with the adjacent drive pattern and different acquisition frame rates; **(D)** Low–pass and comb filtered epileptic EEG after interfered by EIT with the Avis–barber cross drive pattern and different acquisition frame rates.

Randomly selected EEG signals from the CHB-MIT Scalp EEG Database were superimposed with EIT signal under different drive patterns and acquisition frame rate to simulate interfered EEG by EIT. Then the interfered EEG were filtered by low–pass filtering and comb filtering. The filtering results were shown in Figures 8, 9. The raw normal EEG and epileptic EEG were used to compare with the filtered EEG to verify the effectiveness of the two filters. As Figures 8, 9 shown, although there were slight differences between filtered EEG and raw EEG at some time points, there was a strong agreement between the filtered EEG and the raw EEG on the whole, both in the amplitude of the EEG and the trend of the EEG over time. Epileptic waves are the gold standard for an epilepsy diagnosis. The amplitude and duration of EEG waves are important indicators to judge epileptic waves. After low-pass filtering and comb filtering, both high-frequency and low-frequency interference were effectively suppressed, and the epileptic EEG waves could be recognized.

### Correlation analysis

Pearson correlation was used for quantitative analysis of the similarity between raw EEG and filtered EEG. The Pearson correlation coefficient (PCC) is often used as a measure of similarity. The value of PCC is between −1 and 1. The larger the magnitude, the greater the similarity (53). PCCs were given in Table 2. Under the same drive pattern of EIT, the PCC increased slightly with the increase of the acquisition frame rate. At the same acquisition frame rate, different drive patterns had little influence on the similarity. In general, the value of PCC was >0.95, indicating a high degree of similarity between the raw EEG and the filtered EEG, which also proves that the interference of EIT on EEG can be suppressed effectively and the raw EEG can be restored.

**Table 2:** Pearson correlation coefficient between original and filtered EEG.

**Drive pattern**	**Acquisition frame rate (f/s)**	**Correlation in time domain**		**Correlation in frequency domain**	
		**Normal EEG**	**Epileptic EEG**	**Normal EEG**	**Epileptic EEG**
Polar	1	0.9792	0.9552	0.9904	0.9948
	2	0.988	0.9573	0.9929	0.9964
	5	0.9948	0.9592	0.9975	0.998
	10	0.9954	0.9592	0.998	0.998
Avis-Barber Cross	1	0.9791	0.9552	0.9903	0.9948
	2	0.9872	0.9574	0.9924	0.9965
	5	0.9948	0.9592	0.9975	0.998
	10	0.9951	0.9591	0.9981	0.998
Adjacent	1	0.9793	0.9552	0.9905	0.9948
	2	0.9871	0.9574	0.9927	0.9965
	5	0.9947	0.9592	0.9975	0.998
	10	0.9951	0.9592	0.998	0.998

## Discussion

The interference of EIT in EEG during synchronous monitoring was analyzed in this study. It was found that in addition to high frequency interference of 51.2 kHz, there was also periodic low–frequency interference caused by switching of electrode pairs used to inject drive current. The causes and spectral characteristics of low–frequency interference with different drive patterns and acquisition frame rates of EIT were studied for the first time. According to the spectral characteristics of low–frequency interference generated with different drive patterns and acquisition frame rates of EIT, a comb filter was proposed to filter out the low–frequency interference. Our simulation results suggested that a low-pass filter and a comb filter working together could effectively filter out the interference of EIT on EEG in the process of synchronous monitoring. Furthermore, this method was robust for different drive patterns and acquisition frame rates of EIT.

### Low-frequency interference analysis

EIT measurements were obtained by injecting current through an independent current injecting electrode pair and recording the resulting boundary voltages from all remaining electrodes (54). In this study, 16 electrodes were used to inject current and record the boundary voltages. For every independent impedance measurement, a pair of electrodes injected a constant sinusoidal current, and measurements were taken by all remaining electrodes. The sinusoidal current was injected with different sequences of 16 electrodes pairs: [(*i*_0_, *i*_8_), (*i*_1_, *i*_9_),…, (*i*_15_, *i*_7_)] for the polar drive pattern, [(*i*_0_, *i*_1_), (*i*_1_, *i*_2_),…, (*i*_15_, *i*_0_)] for the adjacent drive pattern, and [(*i*_0_, *i*_4_), (*i*_1_, *i*_5_),…, (*i*_15_, *i*_3_)] for the Avis–barber cross drive pattern. After the measurements were taken from all 16 combinations of electrodes during *T* seconds, a single frame data set was formed. In each frame, the current injection electrodes were switched 16 times. As a result, the frequency period of the low–frequency components due to switching of injection current between 16 electrodes pairs was 16 × 1/*T*Hz. As presented in Figure 5, the frequencies of low–frequency interference were 16, 32, 80, and 160 Hz with acquisition frame rate of 1, 2, 5, 10 fps, respectively.

With the acquisition frame rate n, eight spectral peaks within a low–frequency period appeared at the frequency of *n* × (2 × *m* – 1) (*m* = 1, 2, 3,…) under the polar drive pattern, 15 spectral peaks within a low–frequency period appeared at the frequency of *n* × *m* (*m* = 1, 2, 3,… and *m* ≠ 16) under the adjacent drive pattern, and 12 spectral peaks within a low–frequency period appeared at the frequency of *n* × *m* (*m* = 1, 2, 3,… and *m* ≠ 4) under the Avis–barber cross drive pattern. It should be noted that the frequency corresponding to each spectral peak was an integer multiple of the acquisition frame rate.

### Comb filter

The comb filter is a multi–band filter that removes certain frequencies at band nulls from the obtained feature map (55). It should be able to suppress the harmonics associated with the periodic signal and, simultaneously, preserve the signal required by the application. In the field of biomedical signal processing, comb filtering plays an important role in the elimination or extraction of periodic waveforms, such as electrocardiography signal and EEG signals, for improving diagnostic accuracy. Comb filtering has been proven to be useful for suppressing of the gradient artifact from the EEG signal recorded simultaneously with fMRI data (56). The gradient artifact produced by temporally varying magnetic fields associated with the switched gradient waveforms used in MRI (57) is similar to the low–frequency interference produced by the switched excitation electrodes used in EIT. Moreover, simple calculation and computational efficiency make the comb filter suitable for real-time data analysis and filtering in real-time synchronous EIT and EEG monitoring.

In this work, we have demonstrated the effectiveness and robustness of comb filtering in the suppression of low–frequency interference from EIT (Figures 8, 9; Table 2). EEG was restored after low–pass filtering and comb filtering. From the time domain diagram, there was no significant difference between the raw EEG and filtered EEG, and the normal EEG and epileptic EEG could be recognized effectively. Pearson correlation analysis further confirmed the interference of EIT on EEG was effectively suppressed. Furthermore, the design of the comb filter was determined by the acquisition frame rate of EIT, so there was no need to establish the noise template in advance. This avoided the differences between the template and actual artifact waveforms, which is practical to clinical application.

### Influences of drive pattern and acquisition frame rate on filtering effect

In this study, the influences of drive pattern and acquisition frame rate of EIT on the filtering effect were analyzed. The results suggested that when the same acquisition frame rate was used in EIT, different driven patterns had almost no influence on the low–frequency interference filtering effects of the comb filter (the difference was < 0.08 % as shown in Table 2). Low–frequency interference was determined by the acquisition frame rate of EIT. In our simulation analysis, different EIT protocols involving 16 electrodes were used in different drive patterns. When the acquisition frame rate was unchanged, regardless of which drive pattern was used, the spectrum peaks appeared at the frequency points of an integer multiple of the acquisition frame rate. The comb filter was designed based on the amplitude frequency characteristics of low–frequency interference, so the drive pattern had little influence on the filtering effect when the acquisition frame rate remained the same.

**Figure 10:**
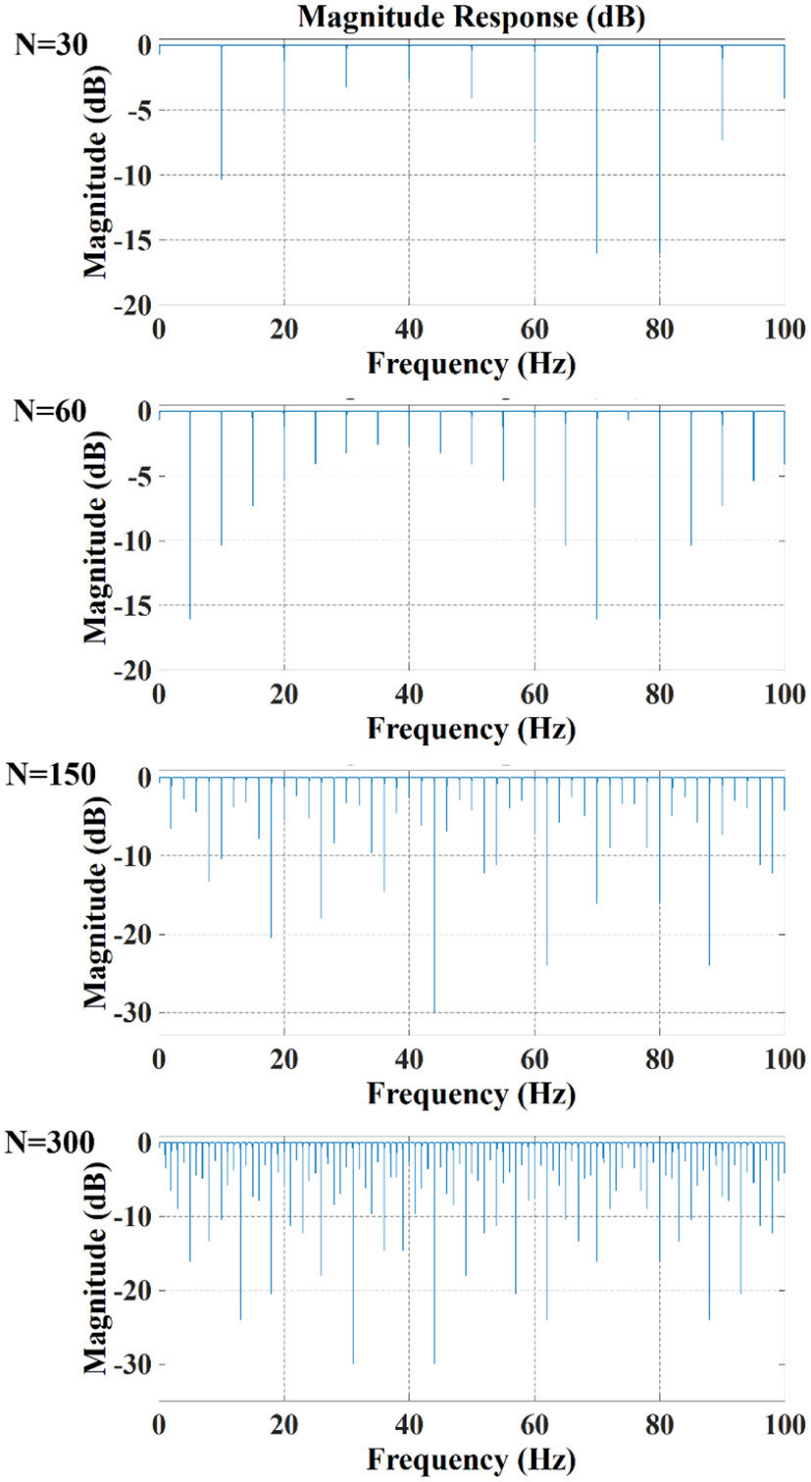
Amplitude response of comb filter with different order N (Bw = 0.3, Apass = 0.05).

In contrast, when the drive pattern remained constant, the filtering effect improved slightly with the increase of the acquisition frame rate. It can be seen from Figures 6, 7 that when the acquisition frame rate decreased, the time required for the 16 electrode pairs to complete switching in a single acquisition increased correspondingly, resulting in the increase of spectrum pulses within the same frequency range. Accordingly, the filter order N of the comb filter, which defines the number of notches (or peaks) in the filter, should be increased to get more notches (Figure 10). Although properly increasing the filter order can improve the filtering effect, a too large order will lead to waveform distortion and complicated calculation.

In addition to the better filtering effect, the higher acquisition frame rate could improve the temporal resolution of the EIT. Epilepsy is a disorder of the nervous system characterized by sudden recurrent loss of consciousness and convulsions. Higher temporal resolution means more information about electrical impedance changes associated with seizures is likely to be displayed. This helps to track electrical impedance changes during epileptic seizures in real time *via* EIT, thereby improving the feasibility of seizure prediction.

## Conclusion

In this study, we found that the combination of a low–pass filter and a comb filter could suppress the interference of EIT on EEG, and the calculation was simple and efficient, as such, this method would be suitable for clinical real-time monitoring of seizures. In addition, this method would be valuable for synchronous monitoring of EEG and other medical electromagnetic devices that work the way EIT does.

In our future work, we will integrate EIT and EEG into a single system to synchronously record EEG and electrical impedance during seizures of animal models. A low–pass filter and a comb filter will be used to attenuate the interference of EIT on EEG in real time. Then filtering parameters will be adjusted to improve the filtering effect.

## Data availability statement

The raw data supporting the conclusions of this article will be made available by the authors, without undue reservation.

## Author contributions

LW, XD, and XS performed study concept and design and provided financial support. WZ performed development of methodology. LW and WZ performed writing, review, and revision of the paper. WZ, RW, WL, GL, and ZJ provided acquisition, analysis, and interpretation of data. All authors read and approved the final paper.

## Funding

This work was partially supported by the National Key Research and Development Program of China (2022YFC2404803), the National Natural Science Foundation of China (NSFC) (51907162), and the Key Basic Research Projects of the Basic Strengthening Plan of the Science and Technology Committee (2019-JCJQ-ZD-115-02).

## Conflict of interest

The authors declare that the research was conducted in the absence of any commercial or financial relationships that could be construed as a potential conflict of interest.

## Publisher's note

All claims expressed in this article are solely those of the authors and do not necessarily represent those of their affiliated organizations, or those of the publisher, the editors and the reviewers. Any product that may be evaluated in this article, or claim that may be made by its manufacturer, is not guaranteed or endorsed by the publisher.
